# The role of antifungals in the management of patients with severe asthma

**DOI:** 10.1186/s13601-020-00353-8

**Published:** 2020-11-06

**Authors:** W. Garth Rapeport, Kazuhiro Ito, David W. Denning

**Affiliations:** 1 Airways Disease, National Heart and Lung Institute, Imperial College, London, SW3 6LY UK; 2 Pulmocide Ltd., 44 Southampton Building, London, WC2A 1AP UK; 3 Manchester Fungal Infection Group (MFIG), Faculty of Biology, Medicine and Health, The University of Manchester, Manchester Academic Health Science Centre, Manchester, M13 9PL UK

**Keywords:** Severe asthma, ABPA, Antifungals, Biologics, Itraconazole, Voriconazole, Aspergillus

## Abstract

In patients with asthma, the inhalation of elevated amounts of fungal spores and hyphae may precipitate the onset of asthma or worsen control to the extent of being life-threatening. Sensitisation to fungi, especially *Aspergillus fumigatus*, is found in 15% to 48% of asthmatics in secondary care and is linked to worse asthma control, hospitalisation, bronchiectasis and fixed airflow obstruction, irrespective of whether allergic bronchopulmonary aspergillosis (ABPA) is diagnosed. ABPA represents a florid response to the presence of *Aspergillus* spp. but up to 70% of patients with severe asthma exhibit sensitisation to different fungi without meeting the diagnostic criteria for ABPA. The presence of persistent endobronchial colonisation with fungi, especially *A. fumigatus*, is linked to significantly higher rates of radiological abnormalities, lower post-bronchodilator FEV1 and significantly less reversibility to short acting bronchodilators. The therapeutic benefit for antifungal intervention in severe asthma is based on the assumption that reductions in airway fungal burden may result in improvements in asthma control, lung function and symptoms (especially cough). This contention is supported by several prospective studies which demonstrate the effectiveness of antifungals for the treatment of ABPA. Significantly, these studies confirm lower toxicity of treatment with azoles versus high dose oral corticosteroid dosing regimens for ABPA. Here we review recent evidence for the role of fungi in the progression of severe asthma and provide recommendations for the use of antifungal agents in patients with severe asthma, airways fungal infection (mycosis) and fungal colonisation. Documenting fungal airways colonisation and sensitisation in those with severe asthma opens up alternative therapy options of antifungal therapy, which may be particularly valuable in low resource settings.

## Background

Severe asthma is a complex heterogenous disease which has been described as “the requirement for high dose inhaled corticosteroids plus a second controller and/or systemic corticosteroids to prevent it from becoming ‘‘uncontrolled’’ or which remains ‘‘uncontrolled’’ despite this therapy” [1].

The natural history of severe asthma is characterised by a long-term decline in lung function, irreversible airways remodelling and increased risk for life-threatening exacerbations. In recent years, significant effort has been directed towards the identification of specific phenotypes which are based on analysis of clinical and biomarker characteristics. Asthma phenotypes may be further subdivided into a small number of endotypes representing distinct disease entities which may benefit from personalised treatment [2]. Fungi are known to play a significant role in allergic airways disease which has been exemplified by allergic bronchopulmonary aspergillosis (ABPA) which is a well described but relatively uncommon endotype [3, 4].

Whilst ABPA represents a florid response to the presence of *Aspergillus* spp., up to 70% of patients with severe asthma exhibit sensitisation to different fungi but do not meet the diagnostic criteria for ABPA [1, 3, 5]. This heterogeneous population has been termed “severe asthma with fungal sensitisation” (SAFS) which is diagnosed with the use of skin prick tests (SPT) and fungal specific IgE responses (Table 1). The conventional diagnostic criteria for ABPA have been subject to revision as a growing recognition that there is broad overlap with a population with fungal sensitisation, airways obstruction and lung tissue damage [3, 4, 6]. In this paper, we provide recommendations on the use of antifungal agents in the patient with SAFS. Fungal nomenclature has changed radically in the last 2 decades and much of the older literature on fungal sensitisation, and indeed many of the currently approved IgE assays and skin test reagents use names that are now obsolete. Additional file 1: Table S1 shows some of the most important allergic fungi and the genera they now belong to and the summary names we use in this paper (see Additional file 1: Table S1).

**Table 1:** Criteria for diagnosis of fungal allergic airways diseases associated with severe asthma and their complications

Disease entity	Clinical criteria	Immunologic	Mycologic	Complications
Allergic bronchopulmonary aspergillosis (ABPA)	1. Asthma or cystic fibrosis^a^ 2. Fleeting or fixed pulmonary opacities on chest radiograph 3. Peripheral eosinophil count > 500 cells/µL	1. Type I *Aspergillus* skin test positive (immediate cutaneous hypersensitivity reaction to Af) or elevated IgE levels against *A. fumigatus*, 2. Elevated total IgE levels more than 1000 IU/mL (unless all other criteria is met, then total IgE levels can be less than 1000 IU/mL) 3. Elevated Aspergillus IgG or precipitating antibodies	None	Bronchiectasis Hyper-attenuated mucous Asthma exacerbations ABPA exacerbations Bronchiectasis exacerbations Fixed airways obstruction Chronic pulmonary aspergillosis Focal pleural based fibrosis areas
Allergic fungal airways disease (AFAD) or Airways Mycosis	Asthma with sensitization and/or inflammation and tissue damage including radiological abnormalities and fixed airways obstruction	Positive immediate skin test (SPT) and fungal specific IgE	Documented (PCR or culture) or presumed	Bronchial wall thickening Bronchiectasis Fixed airways obstruction
Severe asthma with fungal sensitisation (SAFS)	Severe asthma	Positive immediate skin test (SPT) and specific IgE to *Aspergillus fumigatus, Alternaria alternata, Cladosporium herbarum, Penicillium chrysogenum, Candida albicans, Trichophyton mentagrophytes*, or *Botrytis cinereal*	None	Asthma exacerbations Bronchial wall thickening Fixed airways obstruction Chronic pulmonary aspergillosis
*Aspergillus* bronchitis^#^	Non-immunocompromised Major symptoms of cough, breathlessness and sputum production Bronchiectasis common	May have a raised *Aspergillus fumigatus* IgG	Culture or PCR positive for Aspergillus on at least 2 occasions separated in time (to exclude colonisation)	

These entities are not mutually exclusive

^a^Rare cases are described in patients without either of these conditions. # may also be caused by other fungi including *Candida albicans* and *Scedosporium* spp. Reference [5, 6, 84]

## Main text

### Fungal sensitisation, severe asthma and ABPA

Fungal sensitisation to thermotolerant species such as *Aspergillus* has been linked with uncontrolled asthma but the natural history in this population has not been documented in longitudinal studies [3, 5]. A range of radiological abnormalities have been observed including higher than expected rates of bronchiectasis in patients with sensitisation to *Aspergillus* attending severe asthma clinics [7–9]. A recent large cross-sectional study in asthmatics with *Aspergillus* sensitisation documented a range of radiological abnormalities specifically associated with sensitisation to *Aspergillus* but not to environmental fungi such as *Alternaria* and *Cladosporium* suggesting a possible causal relationship [7].

ABPA is described as a progressive disorder with recurrent, infrequent acute episodes that cause successive bronchial damage. Although patients with ABPA are subject to disease progression with long-term remissions, the natural history has not been well described and not all patients progress in a longitudinal fashion [3, 5, 6]. Patients with ABPA usually have moderate or severe asthma, but not all. A number studies have highlighted the role for antifungals for the treatment of ABPA [10–13] (Table 2).

**Table 2:** Overview of antifungal clinical studies (Chronological order)

Antifungal	Dose	Route	target	Fungi	n	Design	Duration	benefits/outcome	Refs.
*Ketoconazole*	400 mg, qd	oral	ABPA, Aspergiloma	S	10	DB	12 M	Af-IgG, symptom score (↓)	Shale [85]
*Itraconazole*	50–400 mg, qd	oral	Aspergillosis Aspergilloma	S	137	Open	11-780D	5 ABPA patients: Symptom (4/5↓) Fungus (3/4↓) cure/improved:60% in IA, 66% in chronic necrotising pulmonary aspergillosis	De Beule [86]
*Inhaled Natamycin*	5 mg, bid	inhaled	ABPA	S	25	DB	50 W	17 patient (9 natamycin, 8 placebo) completed No evidence that natamycin conferred benefit on ABPA	Currie [87]
*Itraconazole*	200 mg, bid	oral	ABPA (CF, asthma)	S C	6	Open	1-6 M (3.9 M mean)	Symptom, tIgE, steroid use (↓),Af-IgG ( →), sputum culture negative in 2/3	Denning [88]
*Itraconazole*	200 mg qd	oral	ABPA	S	12	Open	≥ 6 M	11/12 improvement, blood eosinophil, tIgE (↓), Af precipitins -ve (7/12)	Germaud [89]
*Fluconazole*	100 mg qd	oral	Asthma with dermatophytosis	C	11	DB	5 M + 36 M	bronchial sensitivity to *Trichophyton*, oral steroid use and symptom (↓) PEF(↑)	Ward [68]
*Itraconazole*	200 mg qd	oral	ABPA	S	14	Open	12 M	Lung function (↑), blood esopinophilia, tIgE and steroid use (↓), Af-IgE ( →),	Salez [90]
*Itraconazole*	200 mg bid	oral	ABPA	S	55	DB	16 W	Overall improvement (19% Placebo, 46% ITC), %change on tIgE -60% ITC vs. -44% PLB	Stevens [58]
*Itraconazole*	400 mg qd	oral	ABPA	S	29	DB	16 W	Sputum eosinophil, ECP and serum tIgE/Af-IgG against *A. fumigatus* (↓), Exacerbation requiring oral steroids (↓),%change on tIgE -20% ITC vs. + 1% PLB	Wark [59]
*Itraconazole* *Fluconazole*	200 mg qd 150 mg qd	oral	ABPA	S	44	RS	6 M	ITC > FLU: Better control of asthma symptom, less requirement of reliever/steroid, lesser exacerbation, vs. non-treatment	Rai [91]
*Itraconazole*	200 mg bid	oral	SAFS	S	58	DB	32 W	AQLQ, Rhinitis score, PFT, tIgE (improved vs. Placebo). 60% large improvement. %change on tIgE -27% ITC vs. + 12% PLB	Denning [61]
Itraconazole	100-450 mg qd	oral	ABPA SAFS	S	33	RS	> 6 M	Lung function (↑), tIgE, Af-RAST, eosinophil, steroid use (↓)	Pasqualotto [92]
*Voriconazole* *Posaconazole*	300-600 mg qd 800 mg qd	oral	ABPA, SAFS (Iraconazole-failed)	S	25	Open	≥ 6 M	Clinical response VOR (70% in), POS (78%) after 3 M treatment tIgE, RAST-Af (↓) after ≥ 9 M treatment	Chishimba [13]
*Voriconazole* (EVITA3)	200 mg bid	oral	Af associated asthma	S	56	DB	3 M	no difference on severe exacerbation, QOL, lung function, t or Af-IgE/IgG, blood/sputum eosinophil vs. placebo	Agbetile [62]
Amphotericin B	10 mg bid	nebulised	ABPA, SAFS (Itraconazole/voriconazole failed)	S	21	Open	30D (median) 0–1825D	14% (3/21) Clinical benefit 33% (7/21), failed initial dose due to Bronchospasm 52% (11/21), discontinued within 12 M	Chishimba [11]
Amphotericin B	10 mg bid thrice a week	nebulised	ABPA	S	21	DB	4 M	Frequency of exacerbation (↓ vs. nebulized budesonide), 3 patients, bronchospasm after nebulization of AMB	Ram [12]
*Itraconazole*	200 mg bid	oral	ABPA (acute stage)	S	131	DB	4 M	ITC was effective, but overall efficacy: ITC < prednisolone, side effects ITC < prednisolone The time to the first exacerbation: ITC = prednisolone %change on tIgE -66% ITC vs. 67% PDS	Agarwal [10]
Voriconazole	200 mg bid	oral	ABPA (acute stage)	S	50	unblinded, randomised	4 M	VRC: Exacerbation, IgE, SGRQ(↓), Lung function (↑) But, VRC appears to be as effective as prednisolone	Agarwal [93]
Amphotericin B	10 mg bid	nebulised	Pulmonary Aspergillosis	S	177	RS	4 M to 6Y	Poorly tolerated (66% only) due to increased breathlessness Some patients showed t/Af IgE, Af-IgG(↓)	Otu [60]

ABPA, Allergic Bronchopulmonary Aspergillosis; Af, Aspergillus fumigatus; AMB, Amphotericin B; bid, bis in die (twice daily); C, colonised (culture; PCR or any format of fungal detection); D, day; DB, double blind; ITC, itraconazole; M, month; PDS, prednisolone; POS, posaconazole; PLB, placebo; qd, quaque die (once daily); RS, retrospective; S, sensitised (blood IgE or skin); SAFS, Severe Asthma with Fungal Sensitisation; tIgE, total IgE; VOR, voriconazole; W, week; Y, year

Approximately 5% of the general population, up to 42% of atopic patients, and 70% of patients with severe asthma are sensitised to one or more of approximately 80 different fungi [3, 5]. The prevalence of fungal sensitisation displays wide geographical variation. Exposure to fungi, and/or dampness at home with the presumption of fungal growth in the home or work, can precipitate the development of asthma. This is well documented in infants, children and teenagers in multiple studies [14–16]. Furthermore, in asthmatic children and adults, significant fungal exposure exacerbates asthma, on occasion precipitating hospital admission [16–18]. *Alternaria* sensitisation in childhood in linked to persistent asthma in adulthood [19].

Relatively few severe asthma population studies have been published. A recent study in Sweden of 1006 adults [20] reported 27–32% with sensitisation to moulds with an elevated serum IgE among those with severe asthma. The severe asthma prevalence and fungal sensitisation rates in this study were broadly consistent with data reported in other population-based studies in Denmark, Belgium and Israel [21–25], although lower than in secondary care cohorts the UK [26, 27] and Singapore (66–80%) [28].

The number of adults with severe fungal asthma (encompassing ABPA and SAFS) is large. A global estimate of ABPA based on adult asthma prevalence was 4.7 million [29]. A recent literature review from Africa indicates a 6% asthma prevalence and a pooled estimate of fungal sensitisation of 23.3%, so about 4 million people there with SAFS and/or ABPA [30]. In India, where ABPA is particularly common, the best estimate of ABPA prevalence was 1.38 million with an additional 1 million with SAFS [31]. In Brazil, an estimated 390,500 adults have ABPA and nearly 600,000 have SAFS [32], probably with some overlap in cases. A report from the Global Action Fund for Fungal Infections (GAFFI) related to Latin America put the total number of fungal asthma cases about ~ 1.58 million or 254 per 100,000 [33].

### Persistent lung colonisation with Aspergillus is linked to severe asthma

Investigators have detected high rates of positive sputum culture samples for *A. fumigatus* in SAFS and ABPA populations, with greater than 80% of subjects having at least one positive sputum result at some point [7, 8, 26, 34, 35]. Persistent *Aspergillus* colonisation of the airways has been linked to adverse clinical outcomes which include higher rates of radiological abnormalities, lower post-bronchodilator FEV1, and significantly less reversibility to short acting bronchodilators in patients with a positive sputum fungal culture [8, 34–37]. A heavy burden of fungal colonisation may occur in the absence of IgE sensitisation, a condition which has been called fungal bronchitis. Fungal bronchitis may present with chronic productive cough, loss of asthma control and a decline in lung function and is responsive to treatment with oral azoles [4, 38].

### Diagnosis of fungal allergy in the patient with asthma

The term “Severe Asthma with Fungal Sensitisation” (SAFS) was introduced by Denning et al. in 2006, to describe those patients who have persistent severe asthma (despite standard treatment) and evidence of fungal sensitisation, as defined by positive SPT, or fungus or fungal antigen-specific IgE, and do not meet the criteria for ABPA [3]. The diagnosis of ABPA can be reliably ascertained where the disease is florid but there may be uncertainty with early disease presentation particularly in the absence of bronchiectasis (Table 1). No single test has both good sensitivity and specificity for the diagnosis of ABPA—a definitive diagnosis depends on 8 parameters which include the following: asthma or CF; immediate *Aspergillus* skin prick test positivity, IgE levels > 1000 IU/mL, positive *A. fumigatus* specific IgE (no value specified or studied), *Aspergillus* precipitins (or IgG) detectable, eosinophil count > 1,000 cells/uL, transient pulmonary radiographic opacities, (central) bronchiectasis and high attenuation mucus [4–6]. ABPA is relatively unusual—about 10% of people with asthma associated with IgE sensitisation to *Aspergillus* fulfil all the standard criteria for ABPA [3–5, 39]. There is limited evidence that total IgE is linked to disease severity, but it continues to be widely used as a primary marker of differentiation between ABPA and the diagnosis of fungal sensitisation [40]. This has led to the proposition that a more liberal definition for allergic fungal airways disease should be adopted which removes the requirement to differentiate ABPA for SAFS using total IgE levels as the main discriminator [39, 40].

A positive SPT has value as a screening test for sensitisation, but it is recognised that specific IgE to *A. fumigatus* is the most useful biomarker for the diagnosis of fungal sensitisation [3, 5, 39]. A proposed positive cut-off for *A. fumigatus*-specific IgE (0.35 kUA/L) is two to three times the level of *A. fumigatus*-specific IgE in asthmatics who are already demonstrating positive results on *Aspergillus* skin test [6]. It is important to note that there may be discrepancies between SPT and fungal specific IgE responses which may be explained by batch to batch variations in the production of fungal extracts. Assays for *A. fumigatus* specific IgE are commercially available and might minimize the cross-reaction among different fungal species, which tend to occur when crude extracts of allergen are used. This has led to the recommendation that SPT and fungal specific IgE levels should both be required to confirm the diagnosis of fungal sensitisation, but this ignores the problem of extract variability [40].

Fungal specific IgE sensitisation to *A. fumigatus* has been reported in a population of asthmatics with fixed airflow obstruction and radiological abnormalities [7] which suggests that the sensitisation to thermotolerant filamentous fungi is the major risk factor for the development for lung damage in asthma irrespective of whether the criteria for ABPA are present [39, 40]. To address this population, the concept of allergic fungal airways disease (AFAD) has been proposed where fungal sensitisation occurs in addition to evidence of lung tissue damage, usually documented by radiological abnormalities [3, 7, 39, 40]. A broader term recently introduced is “airways mycosis”, encompassing the upper and lower airways and emphasising the presence of fungi in the airway, driving aberrant immunological responses [41].

To increase diagnostic sensitivity, significant effort has gone into the search for additional markers of disease severity. Numerous allergens from the genus *Aspergillus* have been identified using sera from patients with ABPA [42]. Allergens may be species- or genus-specific, while others are pan-allergens that display cross-reactivity across different fungi [43]. The use of purified natural or recombinant allergens has been used to map allergen sensitisation at a molecular level and has the potential to distinguish genuine sensitisation from sensitisation to cross-reactive allergens from different fungal sources [44], including different recombinant *A. fumigatus* allergens [45]. A commercially available multiplex chip contains more than 100 allergens (ISAC) which enables rapid identification of IgE responses against a broad array of pre-selected antigens [46].

Elevated precipitating antibodies against *A. fumigatus*, have been reported in ABPA and asthmatics with radiological infiltrates which may reflect active disease. *A. fumigatus*-specific precipitins have also been widely used in the diagnosis of ABPA, but their positive rates in cases with ABPA ranged widely [47–49]. The optimal cut-off value of *A. fumigatus* specific IgG measured by ImmunoCAP method has recently been proposed for the diagnosis of ABPA and is not required for SAFS [50]. A recently proposed revision of the criteria for the diagnosis of ABPA using a lower IgE of 417 IU/mL (as in the original definition) has been published with an overall excellent sensitivity of 96% [51]. A lateral flow assay for Aspergillus fumigatus IgG and IgM had a 92% sensitivity and 82–91% specificity [52, 53]. Elevated blood eosinophil levels are found in ABPA and define one treatment-responsive phenotype of asthma but also are commonly encountered in many other disorders. In a recent study, blood eosinophilia was the only inflammatory biomarker to be related to radiological markers of lung damage in asthmatics with fungal sensitisation but is not specific [54].

A recent study using qPCR detection in lower airway samples demonstrated the presence of fungi (*Candida, Penicillium, Aspergillus*) in 86% of adult asthmatics. Plasma and BAL IL‐4, IL‐6, IL‐10, IL‐13 and TNF‐α correlated with fungal presence in BAL. Elevated galactomannan in BAL was identified in a small sub-group but concordance testing for *A. fumigatus* suggests that this may reflect the presence of other fungi. The authors suggest that fungal PCR measured in BAL is a sensitive indicator for the presence of fungi in the lower respiratory tract [55]. Farrant et al. also found high level of PCR signal of *A. fumigatus* in sputum samples from SAFS and ABPA [8]. Furthermore, recent mycobiome analysis [56, 57] revealed fungal colonization in higher proportion of patients with severe asthma including *A. fumigatus* spp. complex and *Candida* spp. and the colonization was increased with corticosteroid treatment.

**Fig. 1:**
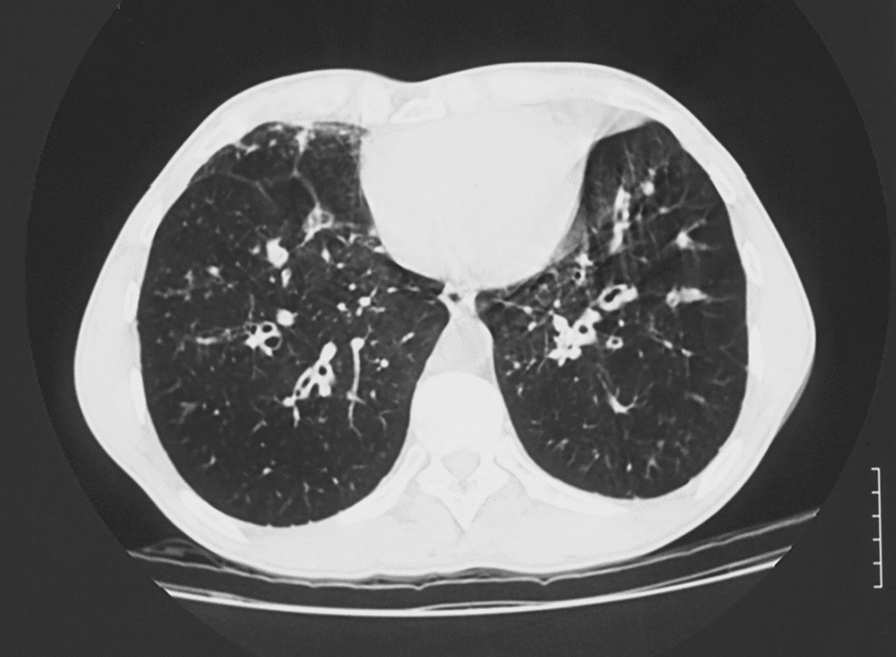
Bronchiectasis complicating ABPA. Bilateral lower lobe bronchiectasis with marked bronchial wall thickening in most bronchi, but not all. Some distal air trapping and some tree in bud appearances

**Fig. 2:**
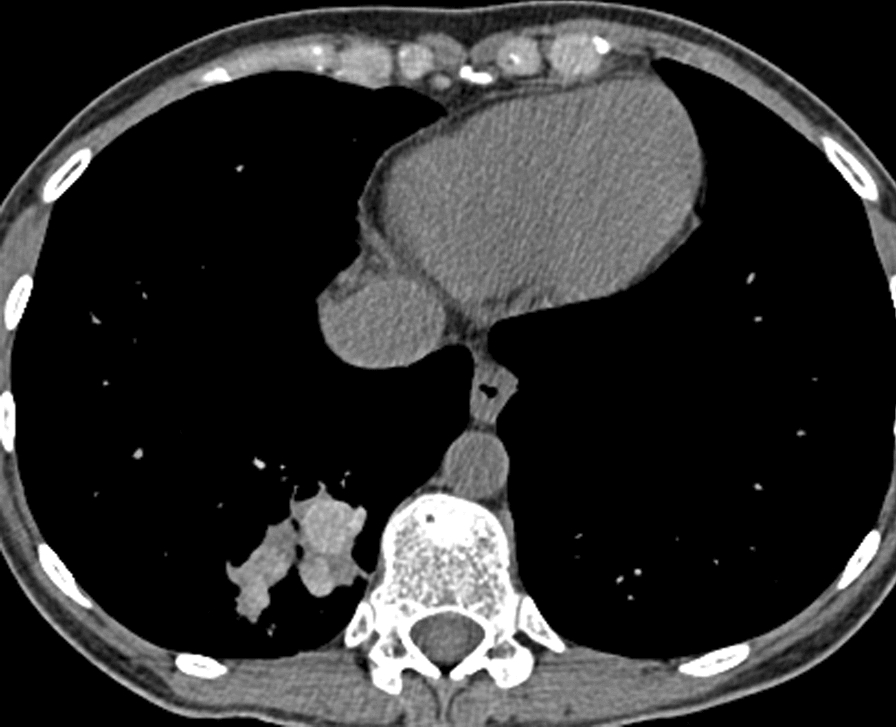
Hyper-attenuated mucous obstructing distal segments of the right lower lobe in ABPA. Woman with asthma who developed ABPA after exposure to a mouldy house (new renovation that leaked). During pregnancy starting coughing black plugs up and had an episode of ‘pneumonia’. CT scan shows rounded opacities posteriorly in the right lung base which are enlarged bronchi full of mucus. On the lung windows, rounded shadows are typical of mucus in bronchi, with some surrounding inflammatory change, on the bone windows (shown here), the mucus in some places appears white than others, consistent with hyper-attenuated mucous (HAM)

A wide range of radiological abnormalities have been reported in ABPA and SAFS [6, 7, 39, 40]. High attenuation mucus (HAM) and central bronchiectasis are recognised characteristics of ABPA [6] (Figs. 1, 2). Minor degrees of bronchiectasis, upper lobe fibrosis, tree in bud and consolidation are the most frequently reported radiological abnormalities in SAFS [7–9]. These data suggest that lung damage is a consequence of *Aspergillus* sensitisation and may represent a disease continuum. ABPA can be effectively treated with antifungal agents [10, 13, 58, 59] (Table 2) and the concept of the disease continuum would support intervention with antifungals as a plausible approach for the prevention of disease progression and lung damage.

### Antifungal treatment in patients with asthma

The rationale for antifungal treatment for asthmatics with allergic fungal airways disease has been based on the adverse association of *Aspergillus* colonisation in the airways with asthma disease severity [7–9, 28, 34].

Relatively few prospective, controlled studies have been undertaken (Table 2) but consistent improvements in asthma control have been reported following treatment with a variety of antifungal agents. Most prospective controlled trials report the use of itraconazole in the treatment of ABPA and SAFS [10, 37, 58, 59].

In the multicentre trial of Stevens et al., 46% of patients with ABPA receiving itraconazole responded compared with 19% of those receiving placebo (p < 0.04) [58]. In the study of Wark et al*.* treatment with itraconazole resulted in normalisation of sputum eosinophilia, eosinophil cationic protein level, and a decrease in serum total IgE level and Aspergillus-specific IgG level [59]. These results suggest an anti-inflammatory benefit of itraconazole in ABPA in asthma patients which is believed to be the consequence of reduced *Aspergillus* antigenic stimulus.

A randomised open label study of 131 treatment-naive patients with ABPA has recently published [10]. Patients were allocated for treatment with itraconazole or prednisolone for 4 months. The primary outcomes included the composite response after 6 weeks, percent decline in IgE after treatment, and exacerbation rates. The composite response at 6 weeks was higher in the prednisolone group but the decline in IgE and exacerbation rates after 1 and 2 years of treatment were similar in both groups. Fungal loads were not measured in the study. The adverse event profile in the steroid group was markedly higher versus the itraconazole treatment [10].

The antifungal treatment benefit in ABPA may not be restricted to itraconazole – in a retrospective study of patients with ABPA and SAFS, clinical response to voriconazole was observed in 12/16 (75%) and in 7/9 (78%) for posaconazole [13]. Further evidence for the benefit of antifungal intervention in ABPA has been evidenced by positive therapeutic benefit with the use of inhaled amphotericin [11, 12, 60]. In a recent prospective study of 21 ABPA patients, authors compared treatment with nebulised amphotericin and budesonide versus budesonide alone [12]. While the study was insufficiently powered to detect differences in immune biomarkers, the number of patients experiencing exacerbations was significantly lower in the antifungal arm compared to the budesonide arm (1/12 [8.3%] vs. 6/9 [66.7%]. The study did not report measurement of fungal load in sputum. A number of retrospective studies have highlighted the high incidence of bronchospasm with inhaled amphotericin which restricts widespread utility in this population [11, 60].

Only two prospective, double blind controlled clinical trials of antifungal therapy in patients with SAFS have been published. An early trial of 8 months of itraconazole (FAST study) reported improvement in quality of life scores [61] and pulmonary function tests whilst a subsequent 3 month study (EVITA3) found that voriconazole was not effective [62].

In the FAST study, fifty-eight patients with severe asthma (BTS level 4 or 5) and receiving high dose inhaled steroids were enrolled and treated with itraconazole 200 mg BD or placebo. Patient inclusion was based on positive RAST and skin prick testing to *Aspergillus fumigatus, Cladosporium, Penicillium* spp*, Candida albicans, Trichophyton* spp.*, Alternaria* and *Botrytis cinerea*. The primary end point in the FAST study was change in the Asthma Quality of Life Questionnaire (AQLQ) score. At 32 weeks, significant improvements were reported in AQLQ score, rhinitis scores, peak expiratory flow rates and total serum IgE in the itraconazole treatment group. The 25 itraconazole subjects who received at least 4 weeks of treatment had individual plasma itraconazole concentrations ranging from 0.65 to 26.1 mg/L with a median of 8.9 mg/L. Reversion to pre-treatment status occurred within 4 months following discontinuation of itraconazole therapy [61].

High rates of Aspergillus colonisation were documented in the EVITA3 study in which 81% of subjects in the placebo group and 84% of subjects in the voriconazole group had at least one positive sputum culture for *A. fumigatus*. However, culture conversion only occurred in 12 patients in the voriconazole treatment group—the remainder were classified possible responses (n = 5) or treatment failures (n = 7) [62]. Unlike the FAST study, no reduction in total IgE levels were observed, but the duration of therapy was only 3 months. This is in contrast to studies with itraconazole where reductions in total IgE have been observed (Table 2).

Voriconazole levels were measured in 24 patients taking placebo and 26 patients taking voriconazole. Sub-therapeutic voriconazole levels (mean level 0.89 [0.2–2.8 μg/ml) were found in a number of patients. The lack of consistent antifungal efficacy may be due to factors including non-linear pharmacokinetics which causes greater inter-individual variability in voriconazole steady-state levels when compared with itraconazole. Itraconazole has greater lipophilicity than voriconazole which results in a greater volume of distribution and this results in a higher plasma: lung ratio than voriconazole [63, 64].

Inhibition by hepatic CYP3A4 by itraconazole may result in increased levels of corticosteroids including budesonide and fluticasone [9, 65–67]. A sub-group of 27 patients in the FAST study was evaluated to address this interaction. Reduced plasma cortisol (< 50 nmol/L) was observed in six of 13 itraconazole-treated patients but not seen in the placebo treated subjects. There was no evidence that patients with the corticosteroid interaction exhibited a greater improvement in asthma symptom scores than those who did not, but numbers were too low to offer conclusive evidence [61].

Therapeutic intervention with antifungal agents has been studied in other populations which include chronic fungal infection of the skin and nails with fungal sensitisation linked with asthma. A small prospective double blind, placebo-controlled study recruited 11 patients with severe asthma, dermatophyte infection (onychomycosis and tinea pedis) and sensitisation with *Trichophyton* [68]. Patients were randomized to treatment with oral fluconazole 100 mg daily or placebo for 5 months followed by open label treatment for an additional 5 months in all patients**.** At study inclusion, all patients had positive immediate skin test responses to *Trichophyton* extract, 9 patients had serum IgE antibodies to *T. tonsurans*, antigen specific IgE antibodies were detected in 8 patients and all patients had measurable IgG and IgG_4_ levels. Sinus CT scans documented extensive disease in 8 patients (4 patients had previously had sinus surgery) reflecting extensive allergic disease. Antifungal treatment was associated with marked improved in asthma symptoms, reduced requirement for corticosteroids and increased peak expiratory flow rates in 9 of 11 patients at the end of the study period. Improvements in asthma control paralleled progressive decrease in signs of fungal infection in the skin and nails suggesting that a reduction in fungal burden accounted for the therapeutic benefit. The response in this study was consistent with previously documented improvements in a similar population treated with griseofulvin [69].

A recent prospective clinical study adopted a new approach [70]. Patients with severe asthma were randomised either to treatment for 4 months with oral itraconazole (n = 50) or to oral prednisolone for 1 month (n = 50) without prior testing for the presence of fungi or markers of fungal sensitisation. Treatment was well tolerated in both groups. Improvements in asthma control (ACT scores), lung function and symptoms were significantly greater in the itraconazole group at 1 month versus the oral prednisolone treatment group. The therapeutic benefit for azole treatment was maintained for the 4 months treatment period. Treatment with itraconazole had no effect on IgE nor on eosinophils leading the authors to conclude that the observed antifungal treatment benefit is most likely to result from reductions in airway fungal burden.

### Proposed treatment algorithm for the asthmatic patient with fungal sensitisation (SAFS)

**Fig. 3:**
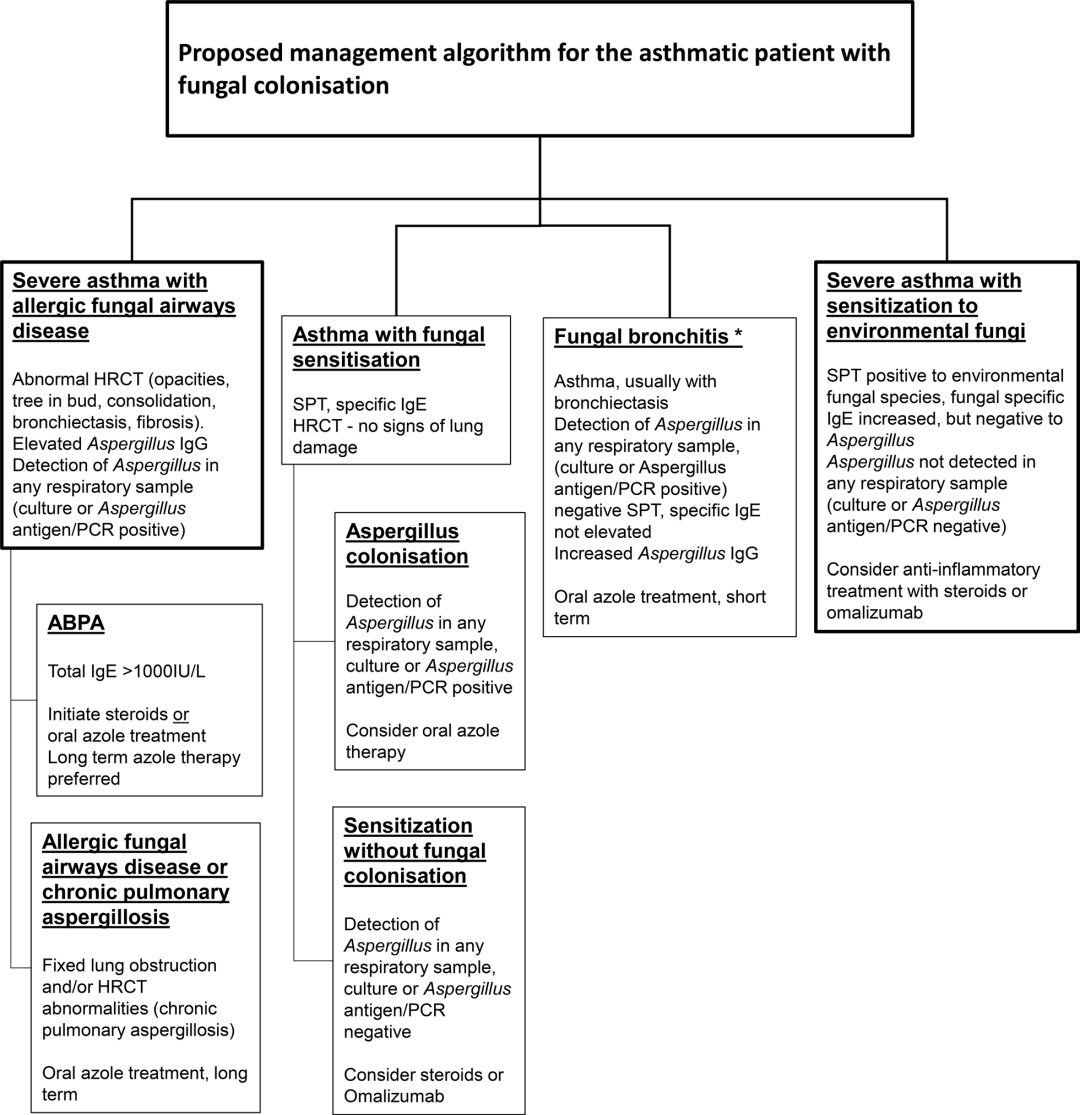
Proposed management algorithm for the asthmatic patient with fungal colonisation. * The diagnosis of fungal bronchitis must be actively sought with at least two positive specimens for fungal culture or PCR (https://www.aspergillus.org.uk/content/aspergillus-bronchitis) [84]

We propose here a treatment algorithm for patients with severe asthma, but first address several key decision points in management (Fig. 3).

- Should SAFS be treated with antifungals in absence of fungal colonisation of the airways?

The therapeutic benefits for antifungal intervention in SAFS assume that elimination of fungal replication in the airways may reduce antigenic stimulus which therefore results in therapeutic improvement. As discussed above, evidence in favour of antifungal therapy has been highlighted in a small number of prospective controlled trials in ABPA and SAFS but none of these have required fungal colonisation as a basis for study entry nor have they studied fungal burden using serial testing [10, 58, 59, 61].

The question is complicated by the insensitivity of standard culture methods for detection of colonisation in sputum samples. Routine clinical laboratory methods using sputum samples (e.g. using the HPA method) report positive detection in only 10% of samples [35]. Using customized separation or high-volume methods, significantly higher rates of *A. fumigatus* have been detected in sputum samples from sensitised asthmatics, but these assays are not routinely available [35]. PCR assays are sensitive and offer semi-quantitative data but only provide indirect evidence for fungal replication. It is also recognised that the fungal burden in ABPA and severe asthma is highly variable within and between individuals [8, 35, 37, 56]. Due to high sample and yield variability, serial testing for fungal colonisation is recommended in the asthmatic with sensitisation to thermotolerant species.

Based on the lack of evidence, it is not possible to recommend antifungal treatment for sensitised asthmatics who have no evidence of fungal colonisation based on at least 3 specimens submitted for fungal culture or Aspergillus PCR. The one exception may be patients with concomitant cutaneous dermatophyte infection, but the treatment should be directed at the skin infection.

- *Is antifungal therapy justified in severe asthmatics with fungal specific sensitisation, fungal colonisation, fixed lung obstruction and/or the presence of radiological abnormalities?*

There is increasing consensus that ABPA and SAFS are overlapping conditions which argues for a pragmatic definition which ignores the requirement for total IgE levels greater than 1000 IU/L [4, 6, 39, 40]. Although systemic steroids are highly efficacious in the management of ABPA, almost 50% of patients relapse when they are tapered and 20–45% become glucocorticoid dependent (currently in India) or become reliant on high dose inhaled steroids [6, 71]. In addition, high dose steroids are associated with a much higher rate of adverse events which argues for the use of antifungals as first choice therapy for long term asthma and ABPA control in this population [10].

The case for antifungal therapy in patients with ABPA is strongly supported by a number of studies [10, 58, 59, 72, 73]. Benefits of antifungals for ABPA and SAFS have identified major steroid reductions and improvement in asthma control as the most important [5].

Given the increasing consensus regarding the overlap of ABPA and severe asthma, fungal sensitisation and radiological features of lung damage, it seems likely that antifungal therapy would offer significant therapeutic benefit to the majority of patients who meet some of the criteria for ABPA irrespective of total IgE levels [4, 6, 39, 40]. The rationale for antifungal therapy is strengthened by evidence for airways colonisation. It should also be noted that existing data supports a pathogenic role for *Aspergillus* in severe asthma, but this has not been established for thermotolerant yeasts such as *Candida albicans* which are frequently encountered colonizing the airways [39, 40].

It is evident that there is high unmet clinical need for improved antifungal therapies for the asthmatic with greater efficacy, safety, tolerability and avoidance of drug interactions (particularly glucocorticoids). The use of inhaled antifungals which enable sustained pulmonary drug concentrations to the site of endobronchial colonisation offers an attractive alternative to the use oral antifungals whilst avoiding the risk of systemic toxicities and adverse drug interactions. The emergence of resistance with the use of oral antifungals is a significant concern and the use of inhaled therapies with maintenance of high local drug concentrations would be expected to reduce this risk.

Four inhaled antifungals are currently in development for the treatment of fungal infections in ABPA and severe asthma or invasive aspergillosis. PUR1900 (Pulmatrix Inc) is a novel dry powder formulation of Itraconazole which has been repurposed for the treatment of adult asthmatics with ABPA [74, 75]. ZP-059 (Zambon Company S.P.A.) and TFF-VORI (TFF pharmaceuticals) are inhaled forms of voriconazole, and both completed phase 1 recently [76, 77]. PC945 is a novel azole compound which has been designed for topical administration, demonstrating a long duration of action and is being studied in asthmatics (ClinicalTrials.gov Identifier: NCT03745196) [78, 79]. Recently, FDA organized a workshop entitled “Addressing Challenges in Inhaled Antifungal Drug Development”, to discuss the challenges and clinical trial design considerations for developing inhaled antifungal drugs [80].

### Economics of severe asthma

Severe asthma is expensive for patients in terms of time off work and inability to fulfil other social responsibilities and for healthcare systems [81]. In the US, sources claim that the relative minority of severe exacerbators account for a disproportionate degree of total direct treatment costs (> 80%), driven by the expense of emergency department or hospital treatment. Patients who have exacerbations face high healthcare costs, with one commentator suggesting that a single exacerbation requiring additional pharmacotherapy (or more aggressive intervention) can more than triple annual treatment costs [44].

This high expense has allowed the development of multiple novel therapies for severe asthma, mostly targeting individual components of the allergic immune cascade and delivered as monoclonal antibodies (see Table 3—product, target, year of licensure). While the economics of these new treatments puts some strain on healthcare systems in high income countries, the return in terms of reduced unscheduled care needs, less time off work and social benefit in terms of improved health are affordable. The same is not true for low- and middle-income countries, where such costs are prohibitive, especially as long-term therapy is required. The conventional model for such expensive products is to wait until they are off patent and less expensive generics become more affordable, at least in middle income countries. With monoclonal therapies, the production and delivery cost of generic biosimilars will not realise such major reductions in price for low resource settings, and only small molecule development targeting the same pathways as the monoclonal agents is likely to (eventually) be affordable.

**Table 3:** Asthma pricing—biologics

Product	Company	Target	Year of market licensing	Price of 6 months treatment		
				USA (USD)	UK (USD)	France (USD)
Xolair (omalizumab)	Roche/Novartis	IgE	2003	21.2 K	17.4 k	20.5 K
Nucala (mepolizumab)	GSK	IL-5	2015	19.2 K	7.2 K	7.3 K
Cinqair/Cinqaero (reslizumab)	Teva	IL-5	2016	17.4 K	8.4 K	n/a
Fasenra (benralizumab)	AstraZeneca / Kyowa Hakko Kirin	IL-5R	2017	19.2 K	16.6 K	14.4 K
Dupixent (dupilumab)	Sanofi-Genzyme Regeneron	IL-4 IL-13	2018	21.1 K	10.8 K	10.4 K

n/a, not applicable

Sources: RedBook Online, accessed Jan 2020 [94]; MIMS Online, accessed Jan 2020 [95]; Akuthota and Weller UpToDate, 2018 [96]

Note: The US price is the wholesale acquisition cost (WAC), which is the manufacturer's list price for a product when sold to wholesalers. The WAC may not represent actual transaction prices and does not include discounts or rebates Assumptions: Exchange rate of £1:$1.30, €1:$1.20

US Prescribing Information and European SPCs; RedBook Online, accessed Feb 2019 [94]; MIMS Online, accessed Feb 2019 [95]

Xolair dose depends on body weight and pre-treatment IgE serum levels. The US price is the wholesale acquisition cost (WAC), which is the manufacturer’s list price for a product when sold to wholesalers. The WAC may not represent actual transaction prices and does not include discounts or rebates Assumptions: (1) average body weight of 75 kg; (2) no sharing of vials between patients; (3) exchange rate of £1:$1.29, XE, com, Feb 2019

In contrast, oral antifungal therapy is available in most countries and is relatively inexpensive compared to biologicals (Table 4). Both itraconazole and voriconazole are listed on the WHO’s Essential Medicines List [82]. For example, 6 months’ treatment cost of oral itraconazole (at 400 mg daily) is $86.4 in Peru, $180 in Thailand, $136.8 in Egypt, $138.6 in Uganda, $153 in Ghana, $243 in Cote d’Ivoire to name a few low price examples [83]. If a mean daily price of under $1.00 for itraconazole could be established in all low and low middle-income countries, then the annual cost (not including local pharmacy dispensing fees which can be very high) would provide therapy at under $400. While this is still too much for most people in the very poorest countries, it is affordable for several billion people.

**Table 4:** US and UK Pricing of Itraconazole

Generic name	Brand name	Assumed dosing regimen	Cost of 6 months treatment	
			US (USD)	UK (USD)
Itraconazole	Generic	200 mg twice-daily	5418	198
	Sporanox		20,547	869

Sources: RedBook Online, accessed Jan 2020 [94]; MIMS Online, accessed Jan 2020 [95], Akuthota and Weller 2018 [96]

The US price is the wholesale acquisition cost (WAC), which is the manufacturer's list price for a product when sold to wholesalers. The WAC may not represent actual transaction prices and does not include discounts or rebates. Assumption: Exchange rate of £1:$1.30ab

## Conclusions

Severe asthma is characterised by difficult to control disease with long-term declines in lung function, irreversible airways remodelling, impaired quality of life and increased risk for life-threatening exacerbations with attendant health cost implications. Amongst the recognised associations with severe asthma, two overlapping syndromes which result from sensitisation to *Aspergillus* and other airborne fungi (ABPA and SAFS). Evidence suggests that persistent non-invasive endobronchial fungal colonisation with *Aspergillus* in severe asthma is directly associated with signs of lung damage (including bronchiectasis, multiple lung opacities, lung fibrosis). Positive therapeutic benefit has been reported with oral antifungal treatment in a number of prospective studies in ABPA. However, there is a paucity of therapeutic of data supporting therapeutic intervention in SAFS and further studies are required to address this question. Currently, the risk benefit relationship for antifungal therapy must be balanced by the need for prolonged therapy, systemic adverse effects, liability to drug interactions and concern over the emergence of resistance. The recognition that the persistent presence of Aspergillus in the respiratory tract is associated with adverse outcomes enables the targeting of the sub-population most likely to benefit from antifungal treatment.

## Supplementary information

**Additional file 1: Table S1.** Fungal nomenclature for common allergenic fungi.

## Data Availability

Not applicable.

## Abbreviations

ABPA: Allergic bronchopulmonary aspergillosis
ABPM: Allergic bronchopulmonary mycosis
CF: Cystic fibrosis
DB: Double blind
HAM: High attenuation mucous
PC: Placebo control
PCR: Polymerase chain reaction
RAST: Radio allegro-sorbent test
SAFS: Severe asthma with fungal sensitisation
SPT: Skin prick tests
