# Supplementary material for: The role of antifungals in the management of patients with severe asthma
Source: Clin Transl Allergy. 2020 Nov 6;10:46. doi: 10.1186/s13601-020-00353-8 (PMC7646070; doi:10.1186/s13601-020-00353-8)
Supplement: Supplementary file 1 — Additional file 1: Table S1. Fungal nomenclature for common allergenic fungi. [file 13601_2020_353_MOESM1_ESM.docx]

**Additional file 1: Table S1.** Fungal nomenclature for common allergenic fungi

| **Historical name** | **Current fungi linked to that name** | **Fungus label used in this paper** |
| --- | --- | --- |
| *Alternaria alternata* | *Alternaria alternata* | *Alternaria* |
| *Alternaria tenuis* | *Alternaria alternata* | *Alternaria* |
| *Aspergillus fumigatus* | *Aspergillus fumigatus* complex | *Aspergillus fumigatus* |
| *Aspergillus flavus* | *Aspergillus flavus* complex | *Aspergillus flavus* |
| *Aspergillus niger* | *Aspergillus niger* complex  *Aspergillus tubingensis* | *Aspergillus niger* |
| *Aspergillus ochraceous* | *Aspergillus ochraceous* complex | *Aspergillus ochraceous* |
| *Candida albicans* | *Candida albicans* complex | *Candida albicans* |
| *Cladosporium herbarum* | *Cladosporium herbarum* complex  *Mycosphaerella* spp.  *Helminthosporium* spp. | *Cladosporium* |
| *Penicillium chrysogenum* | *Penicillium chrysogenum* complex  *Penicillium notatum*  *Penicillium rubens* | *Penicillium* spp. |
| *Trichophyton* spp. | 18 species and several closely related genera | *Trichophyton* spp. |
